# Perioperative complications and cost of posterior decompression with fusion in thoracic spine for ossification of the posterior longitudinal ligament and ossification of the ligamentum flavum -a comparative study using a national inpatient database

**DOI:** 10.1186/s12891-024-07617-5

**Published:** 2024-07-03

**Authors:** Shingo Morishita, Toshitaka Yoshii, Hiroyuki Inose, Takashi Hirai, Kentaro Yamada, Yu Matsukura, Satoru Egawa, Jun Hashimoto, Takuya Takahashi, Takahisa Ogawa, Kiyohide Fushimi

**Affiliations:** 1 Department of Orthopedic Surgery, Tokyo Medical and Dental University Graduate School of Medicine, 1-5-45 Yushima, Bunkyo-ku, Tokyo, 113-8519 Japan; 2 Department of Health Policy and Informatics, Tokyo Medical and Dental University Graduate School of Medicine, 1-5-45 Yushima, Bunkyo-ku, Tokyo, 113-8519 Japan

**Keywords:** Perioperative complications, Costs, Thoracic ossification of the posterior longitudinal ligament, Thoracic ossification of the ligamentum flavum, Posterior decompression with fusion, Diagnosis procedure combination database

## Abstract

**Background:**

Although posterior decompression with fusion (PDF) are effective for treating thoracic myelopathy, surgical treatment has a high risk of various complications. There is currently no information available on the perioperative complications in thoracic ossification of the longitudinal ligament (T-OPLL) and thoracic ossification of the ligamentum flavum (T-OLF). We evaluate the perioperative complication rate and cost between T-OPLL and T-OLF for patients underwent PDF.

**Methods:**

Patients undergoing PDF for T-OPLL and T-OLF from 2012 to 2018 were detected in Japanese nationwide inpatient database. One-to-one propensity score matching between T-OPLL and T-OLF was performed based on patient characteristics and preoperative comorbidities. We examined systemic and local complication rate, reoperation rate, length of hospital stays, costs, discharge destination, and mortality after matching.

**Results:**

In a total of 2,660 patients, 828 pairs of T-OPLL and T-OLF patients were included after matching. The incidence of systemic complications did not differ significantly between the T-OPLL and OLF groups. However, local complications were more frequently occurred in T-OPLL than in T-OLF groups (11.4% vs. 7.7% *P* = 0.012). Transfusion rates was also significantly higher in the T-OPLL group (14.1% vs. 9.4%, *P* = 0.003). T-OPLL group had longer hospital stay (42.2 days vs. 36.2 days, *P* = 0.004) and higher medical costs (USD 32,805 vs. USD 25,134, *P* < 0.001). In both T-OPLL and T-OLF, the occurrence of perioperative complications led to longer hospital stay and higher medical costs. While fewer patients in T-OPLL were discharged home (51.6% vs. 65.1%, *P* < 0.001), patients were transferred to other hospitals more frequently (47.5% vs. 33.5%, *P* = 0.001).

**Conclusion:**

This research identified the perioperative complications of T-OPLL and T-OLF in PDF using a large national database, which revealed that the incidence of local complications was higher in the T-OPLL patients. Perioperative complications resulted in longer hospital stays and higher medical costs.

## Introduction

Thoracic myelopathy is a condition in which motor and sensory of the trunk and lower extremities are impaired due to compression of the thoracic spinal cord [1]. A retrospective study on Japanese patients stated that the causes and frequency of thoracic myelopathy were ossification of the ligamentum flavum (OLF; 64%) and ossification of the posterior longitudinal ligament (OPLL; 16%) [2]. OLF is characterized with ectopic ossification of the ligamentum flavum behind spinal canal and cord, which is common cause of thoracic myelopathy in Japan [3]. Meanwhile, OPLL is a relatively rare disease caused by ectopic ossification of the posterior longitudinal ligament anterior to spinal canal and cord, resulting in severe myelopathy [4, 5]. Because the thoracic spine generally has kyphotic alignment and the spinal cord is vulnerable, myelopathy in thoracic OPLL (T-OPLL) is often more severe than in cervical OPLL [6]. T-OPLL has different characteristics from the cervical OPLL and thoracic OLF (T-OLF).

Once myelopathy develops, the only effective treatment is surgery because conservative therapy is rarely effective for both T-OPLL and T-OLF [7, 8]. Conventional posterior surgical procedure includes laminectomy and posterior decompression with fusion (PDF) using instrumentation [9]. While laminectomy is partially effective for T-OLF, there are a certain number of cases with poor neurological results [8, 9]. Indeed, a multicenter prospective nationwide survey in Japan found that 48.9% of T-OLF were treated with posterior instrumented fusion surgery rather than laminectomy alone [10]. Although PDF is a reasonable procedure with good neurological improvement for both T-OPLL and T-OLF [10, 11], the details of perioperative complications including systemic complications are unclear. Perioperative complication rates may differ between T-OPLL and T-OLF, even when the same posterior instrumented surgery is performed.

In severe diseases such as T-OPLL and T-OLF, the information regarding the risk of perioperative complications is incredibly helpful in informed consent between patient and spinal surgeon. However, few studies have compared the perioperative complication rates of these two diseases in PDF. Though OPLL is relatively common among Asians including Japanese, relatively rare among Westerners. Furthermore, thoracic OPLL occurs even less frequently than cervical OPLL. Therefore, it is generally difficult to obtain enough cases to study the perioperative complication rate in posterior surgery for thoracic OPLL and OLF. This point is overcome by using a large inpatient database covering all of Japan. We examined perioperative complications after PDF for T-OPLL and T-OLF using the Diagnosis Procedure Combination (DPC) database, a large national inpatient database in Japan. In addition, we adjusted patient characteristics using the propensity score matching (PSM) method to minimize the bias that might affect perioperative complication rates.

## Methods

### Data supply

All information for the present study was obtained from the Japanese DPC database from April 1, 2012, to March 31, 2018. A total of more than 1,000 hospitals including 82 university hospitals participate in the Japanese national inpatient database, providing data on approximately 50% of all acute inpatients in Japan (equivalent to about 7 million people) [12, 13]. The database registered the following information: age, sex, body mass index (BMI), smoking, surgical procedure compliant with Japanese original K-code, admission type, emergency transport, hospital type, preoperative comorbidities at admission compliant with International Classification of Diseases, Tenth Revision (ICD-10) codes, perioperative complications after admission compliant with ICD-10 codes, blood transfusions, length of stay, medical costs during hospitalization, discharge destination, and mortality. This research was approved by DPC Study Group [14] and the Ethics Committee of our institution. Since all data were completely anonymized, informed consent was not required from each patient.

### Patient information

Patients diagnosed with T-OPLL and T-OLF (ICD-10 code, M4884) and underwent PDF (K142-2 or K142-3) through a posterior approach were selected from the database. Patients who underwent anterior and posterior combined approached surgeries were excluded. The registered comorbidities at admission were as follows: diabetes mellitus (ICD-10 codes: E10–14), cardiovascular disease (ischemic heart disease: I200, 201, 208–214, 219–221, 228, 229, 238, cardiac failure: I110, I500, 501,509, atrial fibrillation: I48), hypertension (I10, 15), cerebrovascular disease (I614, 619, I630-639), respiratory disease (chronic obstructive pulmonary disease: J441, 448, 449, pneumonia: J13, 14, 150–159, J180-182, 188, 189, J690, J958), Renal disease (N17-19, N289, I120), Hepatic disease (K704, 711, 719, 720, 729, 769), gastrointestinal disease (K250-270, K279, K922), malignancy (C00-97), rheumatoid arthritis (M069), osteoporosis (M800-805, 808–816, 818, and 819), and mental disease (depression: F313-315, 318–323, 328–334, 339, schizophrenia: F200-209).

### Outcomes

We investigated the outcomes between T-OPLL and T-OLF as follows: perioperative complication rates (systemic and local), reoperation rates, length of stay, medical costs during hospitalization (with and without perioperative complications), transfusion, mortality, and discharge destination (home or another hospital). Since the exact amount of blood loss was not directly known, we evaluated as surrogate outcomes by blood transfusion based on previous reports [15, 16]. The analyzed systemic complications between T-OPLL and T-OLF as follows: cardiovascular complications (ischemic heart disease, cardiac failure, atrial fibrillation), cerebrovascular complications, respiratory complications (pneumonia, respiratory failure: J959-961, 969), Renal complications, Hepatic complications, gastrointestinal complications, peripheral vascular complications (deep venous thrombosis: I801, 802, 828, pulmonary embolism: I269), systemic infectious complications (urinary tract infection: N390, T835, sepsis: A394, 400–403, 409–415, 418, 419), and delirium (F050, 051, 059). The analyzed local complications and reoperations as follows: surgical site infection (SSI) (T793, 814), cerebrospinal fluid leakage (G960, 961), hematoma (S064, 241, 341, T093), meningitis (G001-003,008–009, 039, A390, 392), and wound debridement (K-code: K-002).

### Statistics

Patients diagnosed T-OPLL, and patients diagnosed T-OLF were matched using the PSM method. The procedure for PSM is described below. A logistic regression analysis was performed with all known preoperative variables as explanatory variables. Explanatory variables for logistic regression included age, sex, BMI, smoking (yes or no), admission type (scheduled or unscheduled), emergency (yes or no), hospital type (academic or non-academic), and preoperative comorbidities (diabetes mellitus, cardiovascular disease, hypertension, cerebrovascular disease, respiratory disease, renal disease, hepatic disease, gastrointestinal disease, malignancy, rheumatoid arthritis, osteoporosis, mental disease). Next, propensity scores were calculated using caliper width of 0.4 in the model. Finally, 1:1 pair was created between T-OPLL and T-OLF patients whose propensity scores were close using one-to-one nearest neighbor matching method. Only matched patients were included in the analyses. Fisher’s exact test and the Chi-square test were applied to evaluate categorical variables, and the Student’s t test was applied to evaluate continuous variables. We presented all statistical analyses on Stata IC version 16 (StataCorp, College Station, Texas, USA) and assessed *P* < 0.05 to be statistically significant.

## Results

We recognized total 2,660 eligible patients (T-OPLL: 1,256 patients, T-OLF: 1,404 patients) before matching. The patient’s characteristic (Male: 1,556 cases, Female: 1,094 cases) was significantly different between the T-OPLL and T-OLF group before the matching. In the T-OPLL group, there were younger patients (T-OPLL vs. T-OLF: 56.1 years vs. 66.7 years, *P* < 0.001), fewer male (male: 45.4%, *P* < 0.001), higher BMI (29.5 kg/m^2^ vs. 25.9 kg/m^2^), fewer smoking patients (29.4% vs. 34.4%, *P* = 0.009), and more academic hospitals (17.5% vs. 12.7%, *P* < 0.001) (Table 1). In addition, more patients in the T-OPLL group had diabetes mellitus (33.0% vs. 25.0%, *P* < 0.001). In contrast, other preoperative comorbidities were more frequent in the T-OLF group (cardiovascular disease, hypertension renal disease, malignancy, ​and osteoporosis) (Table 1).

**Table 1:** Patient characteristics between T-OPLL and T-OLF before and after matching

	Before Propensity Score-Matching			After Propensity Score-Matching		
	T-OPLL (*N* = 1,256)	T-OLF (*N* = 1,404)	*P* value	T-OPLL (*N* = 828)	T-OLF (*N* = 828)	*P* value
**Age (years)**	56.1 ± 13.7	66.7 ± 12.5	< 0.001***	61.7 ± 12.0	62.0 ± 13.1	0.61
**Sex**			< 0.001***			0.35
Male	571 (45.5%)	995 (70.9%)		470 (56.8%)	451 (54.5%)	
Female	685 (54.5%)	409 (29.1%)		358 (43.2%)	377 (45.5%)	
**BMI (kg/m²)**	29.5 ± 6.9	25.9 ± 4.9	< 0.001***	27.1 ± 5.6	27.1 ± 5.3	0.97
**Smoking**			< 0.009**			0.90
Yes	369 (29.4%)	483 (34.4%)		255 (30.8%)	252 (30.4%)	
No	709 (56.5%)	758 (54.0%)		475 (57.4%)	483 (58.3%)	
Unknown	178 (14.1%)	163 (11.6%)		98 (11.8%)	93 (11.2%)	
**Admission type**			0.26			0.88
Scheduled	1,107 (88.1%)	1,212 (86.3%)		733 (88.5%)	731 (88.3%)	
Unscheduled	149 (11.9%)	191 (13.6%)		95 (11.5%)	97 (11.7%)	
Unknown	0 (0%)	1 (0.1%)		0 (0%)	0 (0%)	
**Emergency transport**			0.64			0.64
Yes	33 (2.6%)	37 (2.6%)		23 (2.8%)	20 (2.4%)	
No	1,223 (97.4%)	1,366 (97.3%)		805 (97.2%)	808 (97.6%)	
Unknown	0 (0%)	1 (0.1%)		0 (0%)	0 (0%)	
**Hospital type**			< 0.001***			0.95
Academic	220 (17.5%)	178 (12.7%)		124 (15.0%)	125 (15.1%)	
Non-academic	1,036 (82.5%)	1,226 (87.3%)		704 (85.0%)	703 (84.9%)	
**Preoperative comorbidities**						
Diabetes mellitus	414 (33.0%)	351 (25.0%)	< 0.001***	238 (28.7%)	234 (28.3%)	0.83
Cardiovascular disease	79 (6.3%)	154 (11.0%)	< 0.001***	64 (7.7%)	61 (7.4%)	0.78
Hypertension	250 (19.9%)	346 (24.6%)	0.003**	178 (21.5%)	184 (22.2%)	0.72
Cerebrovascular disease	14 (1.1%)	28 (2.0%)	0.07	11 (1.3%)	12 (1.5%)	0.83
Respiratory disease	24 (1.9%)	37 (2.6%)	0.21	13 (1.6%)	15 (1.8%)	0.70
Renal disease	31 (2.5%)	55 (3.9%)	0.035*	24 (2.9%)	24 (2.9%)	> 0.99
Hepatic disease	30 (2.4%)	40 (2.9%)	0.46	21 (2.5%)	21 (2.5%)	> 0.99
Gastrointestinal disease	35 (2.8%)	42 (3.0%)	0.75	26 (3.1%)	28 (3.4%)	0.78
Malignancy	11 (0.9%)	29 (2.1%)	0.012*	10 (1.2%)	7 (0.9%)	0.47
Rheumatoid arthritis	7 (0.6%)	18 (1.3%)	0.05	5 (0.6%)	7 (0.9%)	0.56
Osteoporosis	38 (3.0%)	65 (4.6%)	0.032*	31 (3.7%)	38 (4.6%)	0.39
Mental disease	46 (3.7%)	47 (3.4%)	0.66	27 (3.3%)	30 (3.6%)	0.69

Data were presented as n (%) or mean ± SD. Significant values are given as follows. *P < 0.05, **P < 0.01, ***P < 0.001

T-OPLL: thoracic ossification of the posterior longitudinal ligament, T-OLF: thoracic ossification of the ligamentum flavum, SD: standard deviation, BMI: body mass index, ADL: activities of daily living

Details of perioperative systemic complications in the T-OPLL and T-OLF groups after matching are shown in Table 2. After PSM, 828 pairs of T-OPLL and T-OLF (total 1,656 patients) were created. The overall perioperative complication rate including both systemic and local complications was 21.1% in the T-OPLL group and 19.1% in the T-OLF group, with no obvious significant difference. The systemic complication rate was 12.4% in the T-OPLL group and 12.7% in the T-OLF group, with no significant difference (Table 2). Major complications included cardiovascular events in 2.9% of the T-OPLL group and 3.5% of the T-OLF group, and respiratory events in 1.0% of both the T-OPLL and T-OLF groups, with no significant difference. Gastrointestinal events tended to be more common in the T-OPLL group (4.7%) and the T-OLF group (2.8%). (Table 2).

**Table 2:** Systemic complications after matching

	T-OPLL (*N* = 828)	T-OLF (*N* = 828)	*P* value
Total complications	103 (12.4%)	105 (12.7%)	0.88
Cardiovascular complications	24 (2.9%)	29 (3.5%)	0.49
Cerebrovascular complications	5 (0.6%)	3 (0.4%)	0.48
Respiratory complications	9 (1.1%)	9 (1.1%)	> 0.99
Renal complications	3 (0.4%)	4 (0.5%)	0.71
Hepatic complications	2 (0.2%)	2 (0.2%)	> 0.99
Gastrointestinal complications	39 (4.7%)	23 (2.8%)	0.038*
Deep venous thrombosis	14 (1.7%)	21 (2.5%)	0.23
Pulmonary embolism	4 (0.5%)	1 (0.1%)	0.18
Urinary tract infection	13 (1.6%)	9 (1.1%)	0.39
Sepsis	3 (0.4%)	8 (1.0%)	0.13
Delirium	4 (0.5%)	3 (0.4%)	0.71

Data are presented as n (%). Significant values are given as follows. *P < 0.05

T-OPLL: thoracic ossification of the posterior longitudinal ligament, T-OLF: thoracic ossification of the ligamentum flavum

Table 3 demonstrates the local complication rate at the surgical site and associated reoperation rate after matching. Total local complications in the T-OPLL group occurred in 94 of 828 patients (11.4%), which was significantly 1.5 times higher than in the OLF group (7.7%) (*P* = 0.012). In the analysis of local complications, the significantly higher complications were SSI (6.0% vs. 3.9%, *P* = 0.041) and cerebrospinal fluid leakage (1.5% vs. 0.5%, *P* = 0.044). Although the incidence of paralysis tended to be slightly higher in the T-OPLL group, there was no significant difference (1.9% vs. 1.0%, *P* = 0.100) (Table 3).

**Table 3:** Local complications and reoperations after matching

	T-OPLL (*N* = 828)	T-OLF (*N* = 828)	*P* value
Local complications			
Total complications	94 (11.4%)	64 (7.7%)	0.012*
Surgical site infection	50 (6.0%)	32 (3.9%)	0.041*
Cerebrospinal fluid leakage	12 (1.5%)	4 (0.5%)	0.044*
Hematoma	22 (2.7%)	20 (2.4%)	0.76
Paralysis	16 (1.9%)	8 (1.0%)	0.10
Meningitis	2 (0.2%)	3 (0.4%)	0.65
Reoperations			
Total reoperations	54 (6.5%)	40 (4.8%)	0.14
Debridement	45 (5.4%)	38 (4.6%)	0.43
Others	11 (1.3%)	2 (0.2%)	0.012*

Data are presented as n (%). Significant values are given as follows. *P < 0.05

T-OPLL: thoracic ossification of the posterior longitudinal ligament, T-OLF: thoracic ossification of the ligamentum flavum

Table 4 describes the length of hospital stay, medical costs, mortality, and discharge destination for the T-OPLL and T-OLF groups after matching. Compared to the T-OLF group, the T-OPLL group had an average of 6 days longer hospital stay (42.2 days vs. 36.2 days, *P* = 0.004) and approximately USD 7,700 higher medical costs (USD 32,805 vs. USD 25,134, *P* < 0.001). While fewer patients in T-OPLL were discharged home (51.6% vs. 65.1%, *P* < 0.001), more patients were transferred to another hospitals (47.5% vs. 33.5%, *P* = 0.001). Blood transfusions were more frequently performed in the T-OPLL group than in the T-OLF group (14.1% vs. 9.4%, *P* = 0.003). There were no significant differences in mortality (Table 4).

**Table 4:** Length of stay, cost, discharge destination, blood transfusion, and mortality after matching

	T-OPLL (*N* = 828)	T-OLF (*N* = 828)	*P* value
Length of stay (days)	42.2 ± 38.1	36.2 ± 46.8	0.004**
Cost (USD)	32,805 ± 14,579	25,134 ± 16,699	< 0.001***
Discharge destination			0.003**
Home	427 (51.6%)	539 (65.1%)	
Another hospital	393 (47.5%)	277 (33.5%)	
Others	7 (0.8%)	10 (1.2%)	
Unknown	1 (0.1%)	2 (0.2%)	
Blood transfusion	117 (14.1%)	78 (9.4%)	0.003**
Mortality	1 (0.1%)	0 (0%)	0.32

T-OPLL: thoracic ossification of the posterior longitudinal ligament, T-OLF: thoracic ossification of the ligamentum flavum, SD: standard deviation, ADL: activities of daily living

Data were presented as n (%) or mean ± SD. Significant values are given as follows. **P < 0.01, ***P < 0.001

Table 5 compares medical costs between T-OPLL and T-OLF according to the presence or absence of perioperative complications after matching. Medical costs were higher in the T-OPLL group, whether or not total perioperative complications occurred. The difference was approximately USD 6,900 for total complications (*P* = 0.005), and USD 6,000 for no total complications (*P* < 0.001). Medical costs increased in the OPLL group when perioperative complications occurred for both systemic and local complications, but the difference was not significant. Even in the absence of systemic and local complications, medical costs were clearly higher in T-OPLL. The difference was approximately $6,500 (*P* < 0.001) and $6,000 (*P* < 0.001), respectively. (Table 5).

**Table 5:** Cost between T-OPLL and T-OLF with or without complication after matching

	T-OPLL (*N* = 828)	T-OLF (*N* = 828)	
**Total complication (+)**	**T-OPLL (** ***N*** ** = 175)**	**T-OLF (** ***N*** ** = 158)**	***P*** ** value**
**Cost (USD)**	33,306 ± 17,577	26,404 ± 25,984	0.005**
**Total complication (-)**	**T-OPLL (** ***N*** ** = 653)**	**T-OLF (** ***N*** ** = 670)**	***P*** ** value**
**Cost (USD)**	25,508 ± 9,456	19,486 ± 8,284	< 0.001***
**Systemic complication (+)**	**T-OPLL (** ***N*** ** = 103)**	**T-OLF (** ***N*** ** = 105)**	***P*** ** value**
**Cost (USD)**	32,822 ± 14,651	27,794 ± 30,892	0.14
**Systemic complication (-)**	**T-OPLL (** ***N*** ** = 725)**	**T-OLF (** ***N*** ** = 723)**	***P*** ** value**
**Cost (USD)**	26,350 ± 11,441	19,792 ± 8,560	< 0.001***
**Local complication (+)**	**T-OPLL (** ***N*** ** = 94)**	**T-OLF (** ***N*** ** = 64)**	***P*** ** value**
**Cost (USD)**	34,481 ± 19,591	28,346 ± 33,059	0.15
**Local complication (-)**	**T-OPLL (** ***N*** ** = 734)**	**T-OLF (** ***N*** ** = 764)**	***P*** ** value**
**Cost (USD)**	26,218 ± 10,386	20,175 ± 10,569	< 0.001***

Data were presented as mean ± SD. Significant values are given as follows. **P < 0.01, ***P < 0.001

T-OPLL: thoracic ossification of the posterior longitudinal ligament, T-OLF: thoracic ossification of the ligamentum flavum, SD: standard deviation

## Discussion

We analyzed perioperative complication rates after PDF for T-OPLL and T-OLF using the DPC database, a large nationwide inpatient database in Japan. Patient backgrounds such as age and pre-existing medical conditions could influence the occurrence of perioperative complications. We balanced the patient backgrounds of OPLL and OLF using PSM analysis to build a cohort with similar distribution. Multivariate regression analysis generally has a limit to the number of explanatory variables that can be included depending on the number of occurrences of the outcome. Since some outcomes have a small number of occurrences, including too many explanatory variables can skew the results of a multivariate analysis. To investigate perioperative complication rates as accurately as possible, we used PSM analysis to balance the analysis between the two groups. The results revealed a higher incidence of perioperative local complications such as surgical site infection in OPLL than in OLF as well as higher medical costs.

In this study, the incidence of gastrointestinal complications was significantly higher in cases of OPLL compared to cases of OLF. This may be due to the fact that surgery for OPLL is more invasive than that for OLF, involving more segmental levels and bleeding. Indeed, in reports by Imagama [17] and Ando [10], the average number of ossifications with thoracic posterior fusion surgery was approximately 2.7 levels for OPLL and 1.9 levels for OLF. They also reported that the operative time in OPLL and OLF was 415.7 min and 191.4 min, respectively, and the blood loss was 910.3 ml and 251.6 ml, respectively. Posterior surgery for OPLL requires fusion over wider range, making the surgery more invasive. Verhofste et al. indicated that in spinal fusion surgery for patients with cerebral palsy, the incidence of gastrointestinal complications was 3.4 times higher when the estimated blood loss was ≥ 3 ml/kg/level [18]. In another study summarizing gastrointestinal complications after spinal surgery, gastrointestinal complications were observed in 15.8% of patients [19]. According to the authors, significant risk factors for postoperative ileus included anesthesia time and the surgical levels (OR, 1.202; *P* = 0.047). Therefore, surgery for OPLL generally involves more bleeding, more surgical levels, and longer operation time compared to that for OLF, requiring careful attention to perioperative gastrointestinal complications.

We found that the surgery for OPLL also has the higher local complication rates in this study. This may be due to differences in surgical invasiveness including range of decompression with fusion and surgical procedure. Ando et al. reported that posterior surgery for T-OPLL involved decompression of approximately 5.4 vertebrae and fusion of approximately 7.8 vertebrae [20]. A multicenter study also demonstrated that the number of T-OLF was 1.8 in the fusion surgery group and 2.0 in the non-fusion surgery group [10]. Another research reviewing outcomes of posterior surgery for T-OLF found that the average number of operative segments was 2.8 [21]. As stated above, in OLF, the spinal cord can be decompressed with a relatively simple posterior decompression with fusion. However, the range of decompression with fusion, the exact surgical time and details of the procedure were unknown in the current database.

Several previous reports of posterior thoracic spine surgery indicated that the incidence of surgical site infection was slightly higher for OPLL compared to OLF (5.2–10.2% for OPLL [17, 22, 23], 3.6–7.7% for OLF [24, 25]). In addition, thoracic posterior fusion surgery for OPLL and OLF have a higher infection rate than that of cervical and lumbar posterior fusion surgery. (1.5–2.9% for cervical spine [26, 27], 0.3–0.43% for lumbar spine [28]) When performing posterior thoracic spine surgery, surgeons need to understand that the surgical site infection rate is higher than in the cervical or lumbar spine. In addition, blood transfusions were more frequent in the OPLL group than in the OLF group. This finding could suggest that posterior fusion surgery for OPLL is associated with more blood loss compared to that of OLF. Perioperative bleeding [29] and blood transfusions [30, 31] are considered potential risks for surgical site infection. Particularly, blood transfusions can cause immunosuppression which lead to increase the risk of infection not only at the surgical site but also at other sites infection such as urinary tract infections [30, 31]. If blood transfusions are unavoidable due to perioperative bleeding, more caution should be applied because the risk of perioperative infection can be increased.

Regarding length of hospital stay and medical costs, the OPLL group had a significantly longer hospital stay of about 6 days and higher medical costs of about $7,700 than the OLF group. The longer length of stay and higher medical costs may reflect a higher rate of perioperative complications in the OPLL group. Additional analysis focusing on the occurrence of complications showed that perioperative complications in both T-OPLL and T-OLF led to longer hospital stays and higher medical costs (Table 5). Previous studies have shown that higher medical costs were associated with higher perioperative complication rates in various spinal fusion surgeries including thoracic spine [23, 32, 33]. One report examining perioperative complications and medical costs of cervical degenerative diseases including cervical OPLL found that the occurrence of perioperative complications increased the medical costs of approximately $2,000 to $3,000 for any disease, particularly significant for OPLL [34]. Perioperative complications should be contained as much as possible to shorten hospital stays and control hospitalization costs. In addition, as mentioned above, thoracic OPLL provides more opportunities for fusion surgery and wider segment levels of fusion compared to OLF. Extensive fusion procedures such as OPLL may also increase medical costs.

Regarding postoperative course, T-OPLL patients were more frequently transferred to another hospital but were less frequently discharged home than T-OLF patients. As mentioned above, T-OPLL patients had a higher incidence of perioperative complications and a longer hospital stay. Prolonged bed rest after more invasive surgery is prone to serious perioperative complications such as bedsores, pneumonia, and venous thrombosis [35]. Additionally, an epidemiological study focusing on the socioeconomic status of patients undergoing posterior spinal surgery found an association between length of hospital stay and discharge destination [36]. The loss of independence in activity of daily living and extended hospitalization after perioperative complications may reduce the likelihood of discharge home. To ensure good surgical outcomes, the incidence of perioperative complications should be reduced.

The length of hospital stay was somewhat longer in this data. In both the overall, T-OPLL group, and T-OLF group, the length of hospital stay was longer for patients who were transferred to another hospital than that who were discharged home. The reason for the longer hospital stay in Japanese hospitals is due to the public health systemin Japan. The patients with severe neurological disorders such as T-OPLL and T-OLF are fully rehabilitated even in the acute care hospital where the surgery was performed. Adittionally, in Japan, patients suffered from spine disease who cannot be discharged home are often transferred to other hospitals because they have not acquired the adequate ADL to live at home [37]. A well-designed rehabilitation program over time is important for patients to acquire adequate ADL. However, longer hospital stays require more medical resources, the proper allocation of limited medical resources may be important [38]. To prevent chronic bed shortages, we need to predict that patients less likely to be discharged home to ensure smooth transfers.

The study had several limitations. The current nationwide inpatient database lacked several material data that might affect the surgical outcomes, including imaging findings, and detailed surgical information (surgical time, indication, procedure, range of decompression and fusion). Although the combination of OPLL and OLF is often seen in the thoracic spine, this study does not take that into account due to lack of imaging data. The diagnosis was narrowed down to either OPLL or OLF. In addition, pre- and postoperative neurological findings and patient-reported outcome measures were missing from this database. In a previous report, neurologic deterioration immediately after surgery was clearly more common in patients with T-OPLL than that with T-OLF (41.7% vs. 4.6%) [39], which may have influenced the incidence of postoperative complications. Unmeasured confounders may influence the results with the PSM analysis [40]. Since the information contained in the current database is only available during admission, long-term prognosis after discharge, and the length of hospital stay after transfer to another hospital such as convalescent rehabilitation hospital was unknown. As this database does not include laboratory and imaging findings, misdiagnoses and overdiagnosis may arise. However, previous studies highlighted that the diagnostic ability of the DPC database has a sensitivity of about 80% and specificity of about 90% [41].

Despite its limitations, this study has some valuable points. To date, detailed information of perioperative complications in posterior surgery for thoracic OPLL and OLF has not been well described. As the incidence of OPLL is lower in Western countries than in Asia, information obtained from a national database in Japan is considered to be important. This is the first comparative study in thoracic OPLL and OLF, which are similar but different diseases in posterior thoracic spine surgery. We believe that the findings of this study will be useful in providing appropriate informed consent to patients with OPLL and OLF whom surgery is indicated.

## Conclusions

We compared the perioperative complications and medical costs between T-OPLL and T-OLF in PDF using a large national database, which revealed that the incidence of local complications was higher in OPLL patients than in OLF patients. The occurrence of perioperative complications and OPLL cases could lead to longer hospital stays and higher medical costs.

## Data Availability

The datasets generated during and/or analyzed during the current study are available from the corresponding author on reasonable request.

## Abbreviations

T-OPLL: Thoracic ossification of the longitudinal ligament
T-OLF: Thoracic ossification of the ligamentum flavum
PDF: Posterior decompression with fusion
DPC: Diagnosis procedure combination
BMI: Body mass index
ICD-10: International classification of diseases, Ten revision
SSI: Surgical site infection
